# Temporal sampling of root exudates using coated blade-SPME for decoding plant–pathogen interactions

**DOI:** 10.3389/fpls.2025.1616881

**Published:** 2025-09-16

**Authors:** Hodan Halane, Tim McDowell, Sangeeta Dhaubhadel

**Affiliations:** ^1^ London Research and Development Centre, Agriculture and Agri-Food Canada, London, ON, Canada; ^2^ Department of Biology, University of Western Ontario, London, ON, Canada

**Keywords:** root exudate, metabolomics, passive sampling, coated blade-solid phase microextraction, solid phase microextraction, *Pisum sativum*, Aphanomyces root rot, *Aphanomyces euteiches*

## Abstract

Root exudates shape the rhizosphere and mediate plant–microbe interactions, yet traditional sampling techniques often disturb the natural environment. Here, we present the use of coated blade-solid-phase microextraction (CB-SPME) method for passive, non-invasive *in situ* temporal metabolomic profiling of pea (*Pisum sativum*) root exudates during infection by the soil-borne pathogen *Aphanomyces euteiches.* In comparison to previously established extraction techniques, CB-SPME delivered lower absolute recovery but superior reproducibility while maintaining sensitivity. This non-destructive approach preserves rhizosphere integrity, enabling continuous monitoring of dynamic metabolite fluctuations and offers new insights into how root exudate influences plant–microbiome interactions.

## Introduction

In response to biotic and abiotic stress, plants produce and release a wide range of biologically active compounds into the rhizosphere, referred to as root exudates (Baetz and Martinoia, 2014). These compounds are involved in a multitude of functions in the ecological interactions with the rhizosphere’s microbial communities. Root exudates not only mediate beneficial plant–microbe interactions but also play a crucial role in defense against pathogens by releasing antimicrobial compounds and chemical cues that shape rhizosphere community structure. In the context of soilborne pathogens such as *Aphanomyces euteiches*, these exudates can influence infection dynamics by either attracting or repelling motile spores, or by inducing host immune responses (Oburger and Jones, 2018). Due to the complexity of the root system architecture and interactions with the rhizosphere, sampling root exudates *in situ* while not disturbing the host plant’s natural exudation pattern and surrounding microbiome has been challenging. The lack of suitable non-disruptive sampling has significantly impeded progress in root exudation research.

At present, root exudate metabolomic studies frequently employ discrete sampling methods that necessitate the physical removal of root exudate matrices for sampling (Oburger and Jones, 2018). This approach can alter exudation rates and composition, impacting microbial colonization and activity that influences microbial-mediated processes such as nutrient cycling, pathogen suppression, and overall rhizosphere health (Oburger and Jones, 2018; Escolà Casas and Matamoros, 2021; Wu et al., 2023). Several studies have utilized methods such as exudation traps and rhizoboxes in combination with a root exudate collecting tool (SOIL-REC), which aim to mitigate these disruptions by isolating exudates in a controlled environment and allowing more localized sampling (Shi et al., 2011; Oburger et al., 2013; Vranova et al., 2013). The selective sampling of exudation traps, while useful for targeted studies, is inherently limited as it captures only a subset of the root exudate dynamics (Oburger and Jones, 2018). The use of filter papers and other selective barriers can result in incomplete data, as it may miss critical interactions between different parts of the root system or between the roots and a broader range of soil microorganisms. The act of isolating root segments or employing recirculation systems, as seen in the SOIL-REC method, can induce stress in plant root, which can lead to atypical exudate production. Disturbance in exudation patterns can be particularly problematic due to it altering plant-microbe interaction which, in turn, alters the rhizosphere (Vranova et al., 2013; Oburger and Jones, 2018; Escolà Casas and Matamoros, 2021).

Recently, it has been reported that variables like collection time and diurnal changes affect the composition and abundance of metabolites in root exudates (McLaughlin et al., 2023). These findings underscore the importance of passive sampling, as most metabolites become detectable shortly after exudation begins (McLaughlin et al., 2023). While longer sampling durations may enhance signal strength, they could also mask short-term fluctuations. Therefore, continuous monitoring is essential to capture the complete temporal dynamics of root exudation (Fleishman et al., 2022; McLaughlin et al., 2023). Frequent and/or long period discrete sampling, which involves collecting distinct samples at set intervals, can also lead to a malfunctioning ecological conditions of a continuous system (Vranova et al., 2013; Tian et al., 2021; Fleishman et al., 2022). It was previously shown that there is a high degree of spatial heterogeneity in microbial composition within the rhizosphere, even across a single root (Fleishman et al., 2022). This variation is driven by differences in microbial abundance and diversity relative to the distance from the root surface. Traditional discrete sampling methods often fail to capture this spatial complexity within the rhizosphere, resulting in an incomplete understanding of plant-microbe interactions. Their application in soil environments is limited by challenges such as complex matrices, and the need to physically remove the media, which can disrupt the native rhizosphere conditions.

These challenges necessitate the exploration of approaches that offer ecologically relevant sampling of root exudates in a non-disruptive method. To our knowledge, there is an absence of published studies employing such passive sampling methods for the purpose of root exudate within agricultural research and use in small volume sampling. A coated blade for passive sampling has previously been validated in diverse media, including diluted beef extract, where it effectively sampled veterinary drugs within a short immersion time of 15 minutes (Gómez-Ríos and Pawliszyn, 2014; Gómez-Ríos et al., 2018; Khaled et al., 2020; Kasperkiewicz and Pawliszyn, 2021). Furthermore, passive sampling techniques have also been reported in screening for drugs of abuse in biofluids, pesticides in food, and monitoring of pollutants in aqueous environments (Vrana et al., 2005; Koesukwiwat et al., 2010; Boyacı et al., 2014; Renaud et al., 2022).

The use of passive sampling techniques can be used to monitor and improve our understanding of how specific classes of defense metabolites in plants behave during abiotic and biotic stressors. Isoflavonoids, in particular, are a major class of specialized metabolites in legumes that serve both as signaling molecules in symbiosis and as phytoalexins during pathogen attack. However, how the exudation of these compounds changes during infection, and how they might function as early defense signals, remains poorly understood. This knowledge gap motivated our targeted focus on isoflavonoids as a model class of root exudate metabolites in this study. By leveraging its unique properties, here we employed the coated blade-solid-phase microextraction (CB-SPME) technique to study pea root exudates. In hydroponics, where the root zone is fully immersed in liquid, CB-SPME offers a distinct *in situ* advantage by passively capturing exuded metabolites without changing the media composition. Its compatibility with both aqueous and complex matrices makes it a versatile tool across systems. CB-SPME serves as both a sample preparation and metabolite extraction method, while traditional methods like solid-phase extraction (SPE) and liquid-liquid extraction (LLE) that are widely used in metabolomics, are best suited for discrete sampling. Furthermore, due to its high sensitivity and increased selectivity based on coating, CB-SPME passive sample collection is superior to conventional passive samplers such as filter paper. Here we present the performance of CB-SPME and compare it with SPE and LLE. To maintain methodological consistency, all methods involved the use of pea root exudates from hydroponic systems, where roots were uniformly exposed to *Aphanomyces euteiches* zoospores—the causative agent of root rot disease. We report on root exudate metabolite profiles and evaluate the effectiveness of sampling and extraction methods on the relative yield of various compounds, using both a targeted and untargeted metabolomics approach. We also describe the experimental setup and analytical methods used to adopt CB-SPME as a non-invasive, *in situ*, temporal sampling method for root exudates with the aim to provide a comprehensive resource for researchers interested in adopting passive sampling for similar investigations. This non-destructive sampling technique aligns with the ecological relevance required for studying plant-pathogen interactions.

## Methods

**Figure 1:**
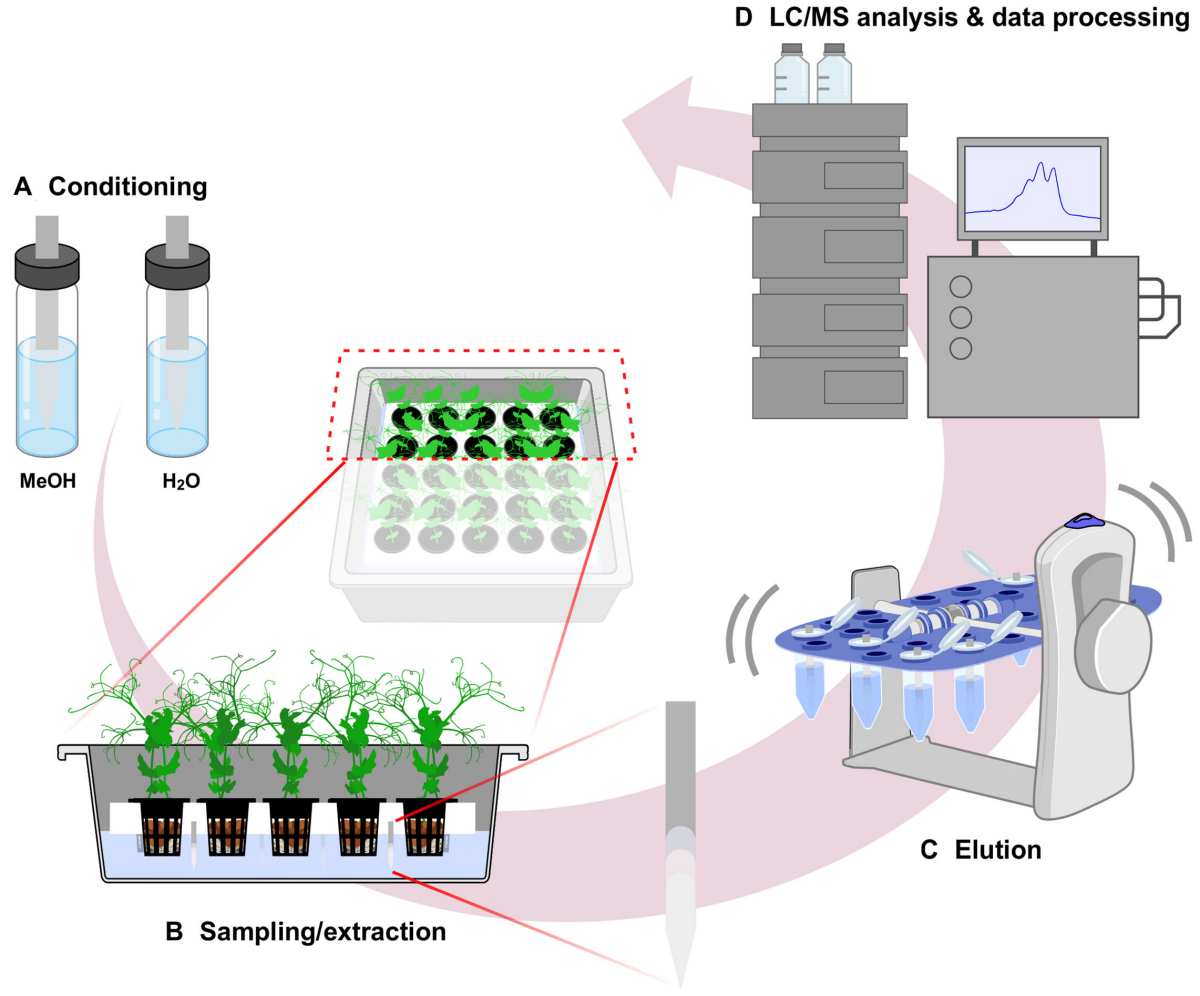
Schematic diagram of CB-SPME technology as passive sampling and extracting method for root exudate. The method contains four steps; A. Conditioning of blades with MeOH followed by H2O wash, B. Passive sampling with blades submerged into the sample matrix, C. Sample elution from blades in MeOH with agitation, D. Sample analysis by LC-MS. CB-SPME, Coated Blade-Solid Phase Microextraction.

A predetermined workflow was established for the use of CB-SPME (Restek, Bellefonte, PA) as both the sample collection and metabolite extraction method (**Figure 1**). Briefly, CB-SPME with HLB-polyacrylonitrile coated blades were preconditioned prior to use. For sampling, blades were directly exposed to the root exudates, allowing *in situ* adsorption of metabolites onto the sorbent coating. Following exposure, blades were immediately transferred to the LC-MS autosampler vials for desorption with methanol and analyzed directly without additional sample preparation.

### Vermiculite-hydroponic-hybrid system

Pea (*Pisum sativum* L.) cultivar CDC Meadow seeds were generously provided by Dr. Syama Chatterton, Lethbridge Research and Development Centre, AAFC. Seeds were planted in pots containing medium sized vermiculate and grown for 14 days in a growth chamber set to 25°C under light intensity 250-400 μmol photons m-^2^s-^1^ and 22°C under dark. The photoperiod was 16:8 hours light:dark cycle with 65% relative humidity. Plants were fertilized with N:P:K 20:20:20 and watered when required. Plantlets were uplifted after 14 days of growth and roots were carefully washed with cold sterile water before being transferred to a hydroponic system. The hydroponic system contained 1.5 L of Mili-Q (MQ) water in 15” x 20” pan covered with extruded polystyrene panel 12.5” x 17.5” with thirty 2” holes drilled (Lebreton et al., 2018). Plantlets were contained in 3” net cup pots with liaflor clay pebbles (hydroton) and placed within the polystyrene panel.

### *A. euteiches* zoospore production

Zoospores were produced following previously described methods with slight modifications (Zitnick-Anderson et al., 2021). Peptone-glucose nutrient broth (2% peptone and 0.5% glucose) was inoculated at room temperature with thirty 3 mm agar sections excised from the advancing edge of 5-day old *A. euteiches* culture growing on corn meal agar and incubated in the dark at room temperature for 3 days. The broth was decanted, and the mycelium was washed twice with mineral salts solution (MSS; 0.026% CaCl_2_·2H_2_O, 0.049% MgSO_4_·7H_2_O, 0.0074% KCl) at 25°C for 1.5 hours per wash. Fresh MSS was then added, and the mycelium was incubated overnight for zoospore production. Zoospore concentration was determined by mixing 1 mL of zoospore suspension with 1 µL of 0.1% aniline blue-lactophenol stain followed by counting using a hemocytometer.

### Discrete and passive root exudate sampling

Two hydroponic systems were set-up with zoospore suspension (~2x10^4^ cells/mL) added to one of the hydroponic systems while the other was kept under mock conditions. Fourteen-day old seedlings were placed into each hydroponic systems. After 24 hours, discrete sampling of root exudate was performed by collecting 5 mL of root exudate media separately for each of the three metabolite extraction methods. The discrete sampling procedure was repeated 5 times independently.

For the passive sampling of pea root exudates to explore the temporal dynamics of metabolite production *in situ*, coated blades were directly placed in hydroponic systems containing either zoospores suspension or under mock solution at the designated sampling points. For example, at 1 day post-infection, three blades (for technical replication) were submerged in the hydroponic media and removed after 6 hours of undisturbed sampling. The same procedure was followed for each sampling point. Sampling was carried over 16 days at nine sampling points (0, 1, 2, 3, 6, 7, 9, 14, 16 days post infection), and the experiment was independently repeated 3 times.

### Metabolite extraction

For SPE, a Waters Oasis HLB 1cc with a 30 mg sorbent SPE cartridge was employed (Waters Canada, Mississauga, ON). SPE cartridges were pre-activated with 100% LCMS grade methanol (ThermoFisher Optima), followed by a wash with water. Samples (4 mL) were loaded, the column was dried for 5 minutes, and analytes were eluted with 100% methanol. The eluates were dried under N_2_ gas and subsequently reconstituted in 90% MeOH.

For LLE, samples were extracted with ethyl acetate, shaken vigorously for 2 minutes, and left to separate for another 2 minutes. The upper layer was collected, and extraction was repeated. The combined extractions were dried under N_2_ gas and then resuspended in 90% MeOH.

For CB-SPME, blades were placed in samples and enrichment occurred for 6 hours before removing the blades. The blades were then placed in 90% MeOH, and agitated on a RotoBot mixer (Thomas Scientific, Swedesboro, NJ) at speed 55 and frequency of 9 for 20 minutes to complete the extraction. Each of the three extraction methods was repeated 3 to 5 times depending on the experiment it was used for.

To evaluate extraction efficiency, a sample containing five isoflavonoids: daidzein (LC laboratories, Woburn, MA), 6α-hydroxymaackiain (MedChem Express, Monmouth junction, NJ), genistein (Caymen Chemical Company, Ann Arbor, MI), formononetin (Toronto Research Chemicals, Toronto, ON), and pisatin (BOC Science, Shirley, NY) of known concentration (100 ng/mL) was prepared for each extraction method and processed using the same extraction procedure described above (CB-SPME, SPE, or LLE) before being subjected to LCMS analysis together with the standards of the same concentration. Relative standard deviation (% RSD) and percent recovery (% RE) was calculated by comparing the metabolite peak area from each extracted sample to the peak area of the standard. 2’-hydroxyformononetin was synthesized in-house at London Research and Development Centre, Agriculture and Agri-Food Canada (AAFC).

All the samples collected with the different extraction methods were analyzed with the same method.

### Liquid chromatography-mass spectrometry

A Q-Exactive Quadrupole Orbitrap mass spectrometer (Thermo Fisher Scientific, USA) with an Agilent 1290 HPLC was used for high resolution mass spectrometry analysis. The HPLC system used a Zorbax Eclipse Plus RRHD C18 column (2.1 x 50 mm, 1.8 μm) to resolve analytes, maintained at 35 C. Samples (5 μL) were run at a flow rate of 0.3 mL min^-1^. Water with 0.1% formic acid (A) and acetonitrile with 0.1% formic acid (B) was used as mobile. Mobile phase B was held at 0% for 0.75 min and increased to 22% over 0.5 min. B was increased to 35% in 2.75 min and to 100% over 3.5 min. It was kept at 100% for 2.5 min before returning to 0% over 30 s. Heated electrospray ionization (HESI) conditions used are as follows: spray voltage, 3.9 kV (ESI+), 3.7 kV (ESI-); capillary temperature, 400 C; probe heater temperature, 450°C; sheath gas, 17 arbitrary units; auxiliary gas, 8 arbitrary units; and S-Lens RF level, 45%. Compounds were detected and monitored using a top 5 data-dependent acquisition (DDA) in positive ionization mode at a full MS spectrum scan between *m/*z 100–1200 at 35,000 resolution, automatic gain control (AGC) target of 3x10^6^, maximum injection time (IT) of 128 ms and intensity threshold of 8.0x10^5^. The MS/MS spectra were collected at 17,500 resolution, AGC target 1x10^6^, max IT 60ms and isolation window of 1.2 *m/z*. Normalized collision energy of 30 was used for the DDA method.

### Computational and statistical analysis

The raw files from LCMS were converted to mzml format with ProteoWizard (Kessner et al., 2008) and imported to R v4.3.2 using the XCMS package (Smith et al., 2006). Features were detected using the centWave method with the prefilters according to Tautenhahn et al. (2008). Feature grouping criteria were established for analytes found in a minimum of 25% of all samples from the three extraction methods. The ‘fillPeaks’ function was applied with default settings.

Target compounds were identified by comparing retention times and *m/z* to authentic standards (**Supplementary Table S1**). When necessary, all features were standardized for untargeted analysis using z-score normalization. To determine features that were significantly different in the three extraction methods, a non-parametric Kruskal-Wallis test was utilized. Two-way analysis of variance (ANOVA) was used to asses significant effects between the extraction methods on their effect on various metabolite levels in samples subjected to two treatments. In the case for the *in situ* root exudate analysis, a two-way ANOVA was employed to assess significance between the treatments. A Tukey’s test was used to conduct *post hoc* comparisons. All data analysis were preformed using R v4.3.2 through RStudio v2023.6.2.0 (Rstudio, 2023).

## Results

### Metabolite recovery using SPE, LLE and CB-SPME sampling methods

To evaluate if the efficiency of the CB-SPME method is comparable to SPE and LLE, a metabolite recovery experiment was conducted. Metabolite recovery was higher in the discrete sampling methods where the entire aqueous media was either extracted with organic solvent (LLE) or passed through a SPE cartridge compared to CB-SPME which was placed in the aqueous media to passively sample (**Table 1**). The mean recovery rate of analytes was 113 ± 32%, 104 ± 37% and 34 ± 11% for LLE, SPE and CB-SPME, respectively. CB-SPME exhibited consistent yet lower recoveries across all metabolites, ranging from 32–37% recovery. Despite this, the % RSD values for CB-SPME remained significantly lower, ranging from 8–15%, highlighting its reproducibility and reliability as an extraction technique (**Table 1**). In contrast, SPE and LLE demonstrated higher % RE values, with some recoveries exceeding practical limits (e.g., 195% for genistein in SPE and 159% for daidzein in LLE). Additionally, the variability for SPE (% RSD: 11–63%) and LLE (% RSD: 21–44%) were considerably higher than CB-SPME (**Table 1**). In addition to reproducibility, CB-SPME also offered the shortest average processing time per sample at 13.5 mins, compared to 85 mins for LLE and 95 mins for SPE (**Table 2**).

**Table 1:** Comparison of metabolite extraction methods on their recovery of target compounds.

Targeted compounds	% RE			% RSD		
	CB-SPME	SPE	LLE	CB-SPME	SPE	LLE
6a-Hydroxymaackiain	32%	56%	142%	15%	63%	39%
Pisatin	37%	108%	94%	9%	11%	44%
Daidzein	34%	68%	159%	13%	33%	21%
Formononentin	33%	94%	80%	8%	37%	30%
Genistein	35%	195%	90%	8%	39%	29%

% RE, Percent Recovery; % RSD, Percent Relative Standard Deviation.

**Table 2:** Per sample average processing time using the different extraction methods.

Metabolite extraction method	Time (min)					Total time
	Conditioning	Sample prep/load	Washing	Elution	Drying-reconstitution	
CB-SPME	1.5	2	NR	10	NR	13.5
LLE	NR	25	NR	NR	60	85
SPE	10	15	5	5	60	95

Extraction time determined by time needed to process 6 samples and calculated for a single sample. NR, Not Required.

### Investigating the metabolite profiles of root exudate using discrete sampling methods

**Figure 2:**
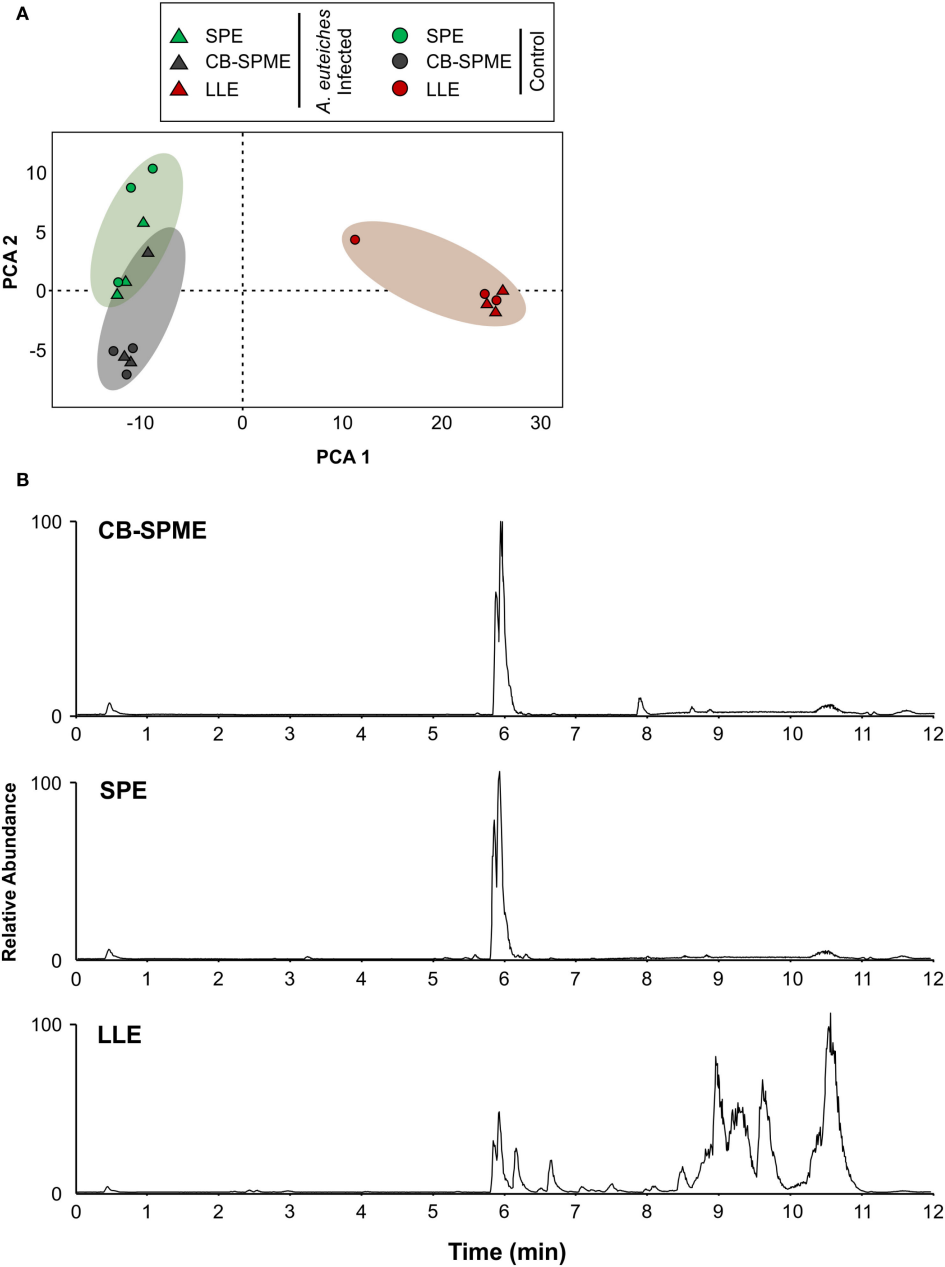
Metabolite profiles of pea root exudates from 24 h *A. euteiches* infected pea roots using three extraction methods. **(A)** PCA plot showing relationship between the effects of using three extraction methods on root exudate metabolites, PCA 1 = 75.58%; PCA 2 = 6.74%. **(B)** Base peak chromatograms of *A. euteiches* infected root exudates collected via a) CB-SPME b) SPE c) LLE. Chromatograms were generated from an Agilent 1290 UHPLC couple to a Thermo Orbitrap Q-Exactive. SPE, Solid-Phase Extraction; LLE, Liquid-Liquid Extraction; CB-SPME, Coated Blade-Solid Phase Microextraction.

We evaluated the three methods on metabolite profiling of root exudate from pea seedlings infected with *A. euteiches* using discrete sampling method that involves collecting samples at a specific regular intervals. The metabolite profiles resulting from each of the three methods were compared via an non-targeted LCMS analysis (**Figure 2**). A total of 302 molecular features were detected in all samples —spanning all three extraction methods (CB-SPME, SPE, and LLE), both treatments (mock and infected), and all biological replicates—using XCMS. Statistical analysis using the Kruskal-Wallis test revealed a significant differential distribution of these features in LLE compared to SPE and CB-SPME (*p* < 0.05). All features were employed in a principle component analysis (PCA) to determine metabolite profiles by extraction method. As shown in **Figure 2A**, different methods showed distinct metabolite clusters, emphasizing method-specific clustering. A clear sample clustering was also shown between the samples extracted with CB-SPME and SPE compared to those extracted with LLE. The base peak chromatograms for CB-SPME and SPE showed similar peak profiles, with comparable peak intensities and retention times, whereas the LLE extracted sample showed an abundant amount of compounds eluting in later retention time (**Figure 2B**).

**Figure 3:**
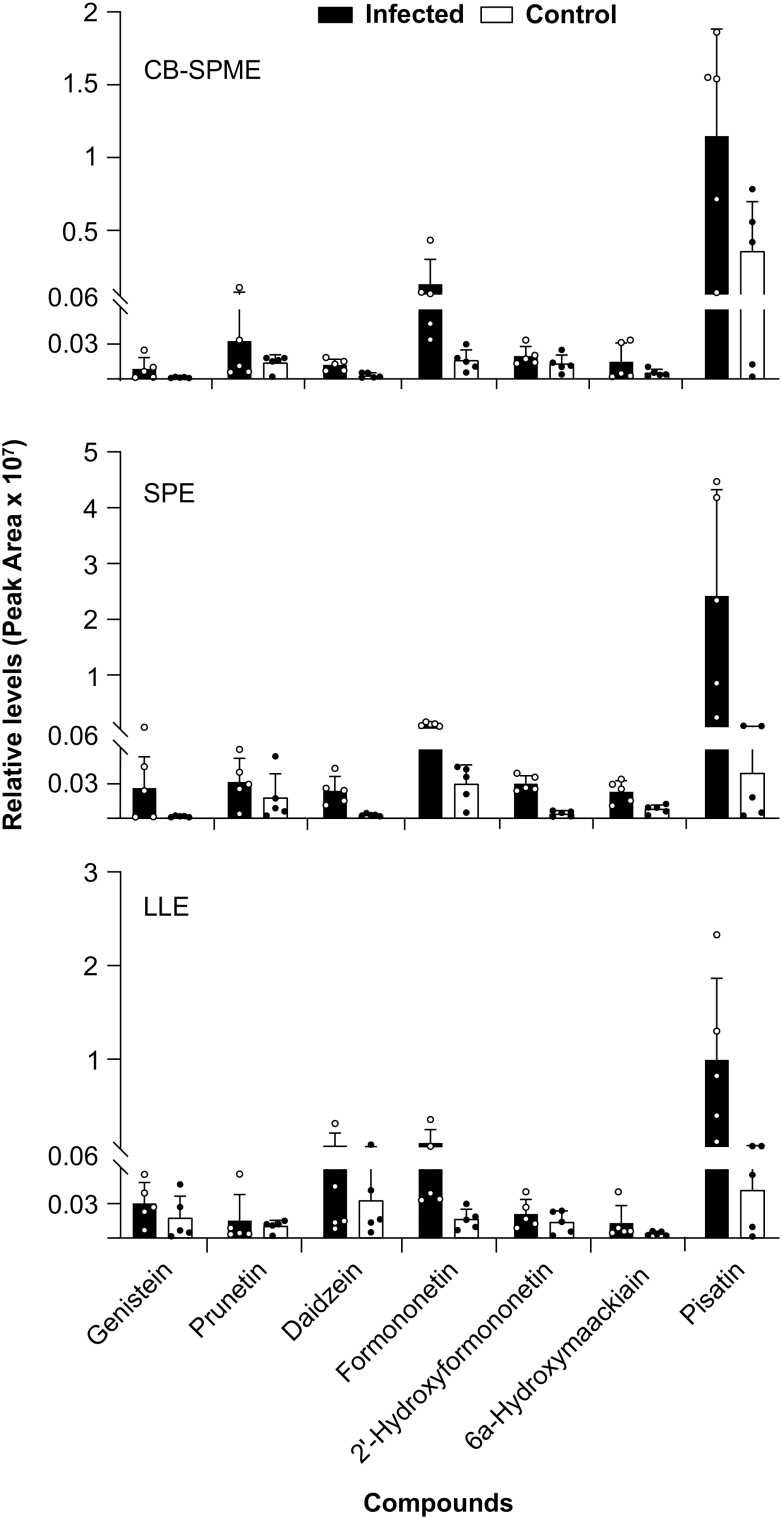
Effect of three extraction methods on the yield of various isoflavonoids. The error bars represent the standard deviation of five biological replicates. SPE, Solid-Phase Extraction; LLE, Liquid-Liquid Extraction; CB-SPME, Coated Blade-Solid Phase Microextraction.

Using targeted analysis, the detection of seven isoflavonoids across the three sampling methods were examined (**Figure 3**). The results revealed that treatment had a highly significant effect on isoflavonoid levels (*p* < 0.0001), indicating that treatment (control vs. infected) significantly influences isoflavonoid concentrations (**Supplementary Table S2**). However, the choice of extraction method did not significantly affect isoflavonoid levels (*p*=0.9901). There was significant variability among different isoflavonoids (*p* < 0.0001). Notably, the interaction between treatment and isoflavonoids was highly significant (*p*=0.0001), suggesting that the impact of treatment varies for different isoflavonoids. In contrast, the interactions between treatment and extraction methods (*p*=0.1340), methods and isoflavonoids (*p*=0.5344), and the three-way interaction between treatment, methods, and isoflavonoids (*p*=0.2214) were not significant. While the treatment group significantly affects isoflavonoid levels, the extraction method itself does not have a significant impact. All three methods did not consistently identify the same isoflavonoids as significantly different following infection, however, CB-SPME and SPE showed a closer alignment in their results (**Figure 3**).

### Temporal root exudate profile of *P. sativum* following infection

**Figure 4:**
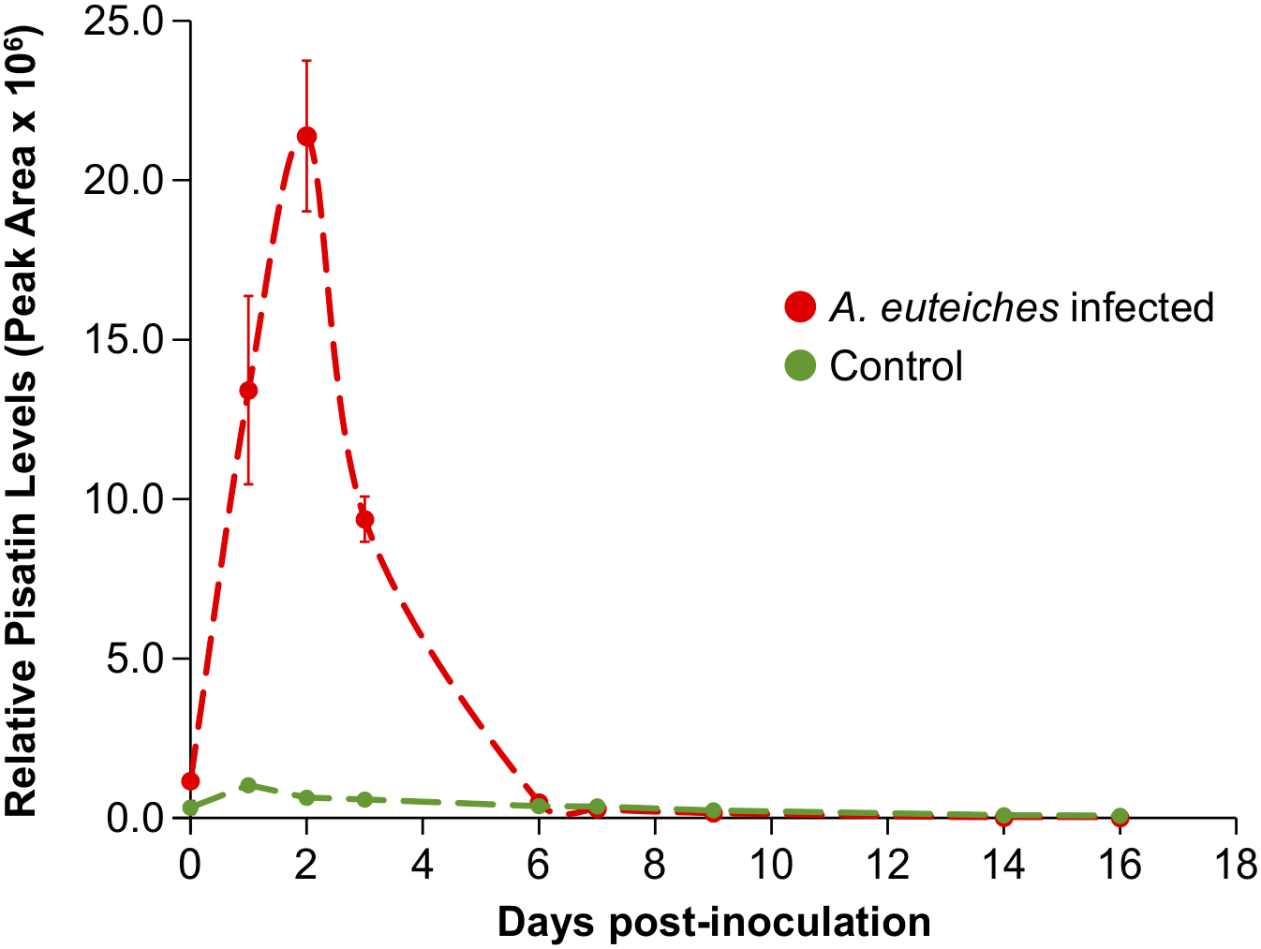
Temporal changes in pisatin levels within pea root exudate in response to different treatments. Passive sampling of metabolites in root exudate achieved via CB-SPME. The error bars represent the standard deviation of three biological replicates and three technical replicates for each biological replicate.

Given the comparable performance of CB-SPME to SPE in extracting metabolites, we selected CB-SPME for its ability to analyze pea root exudates *in situ* without disturbing the rhizosphere. This approach is particularly advantageous for time-course studies that require frequent sampling of root exudate media. Pisatin levels were almost below detection limits in both control and *A. euteiches* infected samples at initial period with an influx being observed in the infected samples at 1 day post-infection compared to non-infected (*p <* 0.05, **Figure 4**). Infected root exudate samples continued to show increasing levels of pisatin, with the maximum peak area observed at 2 days post-infection. At 6 days post-infection, both treatments returned to similar levels of pisatin as 1 day post-infection (**Figure 4**). Pisatin was found at trace levels for the remaining sampling points after 6 days post-infection. A significant difference in pisatin levels between treatment types and across the infection time points was observed (two-way ANOVA; F=11.274, *p* < 0.05, **Figure 4**).

## Discussion

Root exudate are traditionally sampled via discrete sampling method where portions of the surrounding media are removed at fixed intervals. This approach disrupts the rhizosphere’s native conditions and may alter metabolite profiles or microbe-root interactions. The challenges of accurately measuring root exudates is also tied to their dynamic nature and rapid turnover rates (Jing et al., 2023; Lei et al., 2023). Therefore, non-invasive and accurate measurement methods for root exudates are essential to preserve rhizosphere integrity and reliability in results. In this study, we assessed the metabolite profile of root exudates using three different extraction methods via discrete sampling, and then employed an *in situ* root exudate passive sampling technique that removes the disruptive aspects associated with discrete sampling. The extraction efficiency results showed CB-SPME to have lower percent recovery than LLE and SPE (**Table 1**), yet demonstrated superior reproducibility and lower variability across replicates (%RSD), highlighting its consistency and accuracy (**Table 1**). In both SPE and LLE extraction processes, the samples are actively forced through a column containing a sorbent (in SPE) or mixed with a solvent (in LLE), which selectively binds or dissolves desired compounds. This active process enhances interface interactions, often leading to higher recoveries of target compounds (Alvarez et al., 2004; Renaud et al., 2022; Tadić et al., 2022). However, this forced interactions with the sample matrix also introduces greater variability, as reflected in the higher % RSD values for SPE and LLE (**Table 1**). Conversely, the passive nature of CB-SPME, where analyte adsorption depends on natural diffusion at the sampling interface, results in a slightly reduced recovery efficiency compared to SPE and LLE. This passive sampling approach avoids the need for active interactions with the matrix, leading to significantly lower % RSD values across all metabolites, highlighting the method’s reproducibility and reliability. Additionally, the inflated recoveries observed for some metabolites with SPE (e.g., 195% for genistein) and LLE (e.g., 159% for daidzein) suggest matrix effects or co-extraction of interfering compounds, which may compromise the accuracy of quantification (Moreno-González et al., 2017; Cortese et al., 2020; Williams et al., 2023). In contrast, CB-SPME’s consistent recovery rates, despite being lower, offer a more controlled and reproducible extraction process, particularly beneficial for studies focusing on relative metabolite profiling over time. An additional notable advantage of CB-SPME is its ability to use multiple blades simultaneously, in contrast to one-by-one sample processing. CB-SPME enables faster sample preparation and extraction (**Table 2**) while reducing solvent use, making it a more sustainable alternative aligned with green chemistry principles (Vrana et al., 2005; Gómez-Ríos et al., 2018; Kasperkiewicz and Pawliszyn, 2021). Consistent with these performance metrics, both the PCA plot (**Figure 2A**) and the base-peak chromatograms (**Figure 2B**) show that CB-SPME and SPE share highly similar metabolite fingerprints, whereas LLE yields a distinct profile that may reflect the method’s broader affinity for hydrophobic or late-eluting compounds. While CB-SPME showed lower absolute recovery in standard solutions, its metabolite profile closely aligned with that of SPE in real root exudate samples (**Figures 2A, B**). This is likely due to both methods using similar sorbent chemistries, which select for overlapping compound classes. Thus, despite lower yield, CB-SPME reliably captures a representative and reproducible profile, supporting its use for *in situ* biological sampling.

In root exudate, (iso)flavonoids, play a crucial role in plant-microbe interactions (Dhaubhadel et al., 2003; Baerson and Rimando, 2007; Cesco et al., 2010; Tian et al., 2021). Our targeted metabolomics using discrete sampling to investigate the effect of the three methods on the yield of various isoflavonoids in root exudate showed CB-SPME method to have equivalent detection and sensitivity compared to SPE and LLE (**Figure 3**). Notably, their increased abundance in *A. euteiches*-infected samples highlights their role as stress-responsive defense compounds, consistent with previous findings identifying isoflavonoids as reliable indicators of plant immune responses (Ayabe et al., 2010; Shin et al., 2014).

Discrete sampling introduces bias by capturing only a specific moment in the dynamic process of root exudation, potentially missing the temporal variability and full spectrum of metabolites present (Vranova et al., 2013; Escolà Casas and Matamoros, 2021). In contrast, several studies have implemented passive sampling, primarily in aquatic environments, and found it to be superior, identifying more compounds with better isotopic pattern matches, indicating higher contaminant concentrations in extracts (Alvarez et al., 2004; Vrana et al., 2005; Lissalde et al., 2011; Tadić et al., 2022). While the use of passive sampling in the study of root exudates is a relatively new approach, one study utilized SPME fiber for passive sampling of root volatiles from glasshouse-grown broccoli *in situ* (Deasy et al., 2016). However, the insertion of SPME fibers into the vicinity of growing roots may perturb root behavior and alter the soil’s physicochemical properties. This is further complicated by the fibers’ fragility and susceptibility to damage during collection. The use of hydroponic systems allows for direct access to exudates without soil interference, minimizing root disturbance and enhancing the accuracy of studying root exudate dynamics (Strehmel et al., 2014). This approach preserves the rhizosphere environment while enabling precise temporal analysis of metabolites.

Isoflavonoid pisatin, being a phytoalexin, is typically produced by pea plants as a part of their immediate defense mechanism against pathogens (Borejsza-Wysocki et al., 1997; George and Vanetten, 2001; Wu and Vanetten, 2004; Cannesan et al., 2011). The initial peak of pisatin observed at 2 days post-infection suggests an active and robust defense response (**Figure 4**), while the subsequent decline in levels could indicate a potential successful suppression of the pathogen, leading to reduced production of the phytoalexin, or an adaptation of the pathogen to the plant’s defense mechanisms (Miao and Vanetten, 1992; George and Vanetten, 2001). Pisatin accumulation monitored in this study illustrates CB-SPME’s utility in real-time, *in situ* monitoring of defense metabolite fluctuations, capturing biologically relevant temporal data. Notably, while our experiment was conducted in a hydroponic setup, the CB-SPME platform, as previously shown is compatible with both liquid and solid-phase systems. Thus, allowing for expansion to soil and its future applicability for root exudate monitoring in more natural environments.

The passive nature of CB-SPME has proved to be very effective in our temporal targeted metabolomic analysis, opening doors for further root-specific research. This integrated approach minimizes sample handling, reduces the risk of metabolite loss or degradation, and enhances reproducibility in metabolomic profiling. Importantly, it enables detailed investigation of plant–pathogen interactions under conditions that preserve the natural rhizosphere and minimize experimental disturbances. These insights have the potential to lead to breakthroughs in understanding how plants responds to pathogens at the molecular level, and the development of crops that are more resistant to diseases.

## Data Availability

The original contributions presented in the study are included in the article/**Supplementary Material**. Further inquiries can be directed to the corresponding author.
